# Supplementary material for: Identification of potential inhibitors of the main protease from feline infectious peritonitis virus using molecular docking and dynamic simulation approaches
Source: PeerJ. 2025 Jul 30;13:e19744. doi: 10.7717/peerj.19744 (PMC12317694; doi:10.7717/peerj.19744)

**Supplementary tables and figures**

**Table S1. ADMET findings of selected nucleoside analogs and precursors**

| S.N. | Compounds | Solubility level | BBB level | CYP2D6 prediction | Hepatoxicity prediction | Absorption level | PPB  prediction | TOPTAK AMES Score |
| --- | --- | --- | --- | --- | --- | --- | --- | --- |
| 1 | *N3 | -3.652 | 4 | -6.42208 | -7.21011 | 3 | -4.1131 | -25.9284 |
| 2 | 6-Azauridine | 0.586 | 4 | -2.3073 | 3.3959 | 3 | -19.4638 | -0.192769 |
| 3 | Baricitinib | -2.554 | 4 | -12.21 | 6.05048 | 0 | -5.93927 | -1.0869 |
| 4 | favipiravir | -0.041 | 4 | -3.89988 | -0.0946274 | 1 | -13.4262 | -3.07628 |
| 5 | Galidesivir | -0.884 | 4 | -0.67024 | -1.24175 | 2 | -16.7549 | 0.34463 |
| 6 | Gemcitabine | -0.844 | 4 | -4.4137 | -3.57256 | 1 | -18.8277 | -10.1036 |
| 7 | GS-441524 | -0.861 | 4 | -5.53172 | 1.05878 | 2 | -13.6067 | -4.34688 |
| 8 | Methotrexate | -4.018 | 4 | -8.43529 | 23.9597 | 3 | -38.845 | -66.5735 |
| 9 | Mizoribine | 0.546 | 4 | -4.03121 | -0.645759 | 3 | -21.9844 | -4.43329 |
| 10 | Molnupiravir | -0.633 | 4 | -4.78409 | -0.218309 | 2 | -20.3901 | -15.7176 |
| 11 | Oxipurinol | -0.65 | 3 | -6.27898 | -0.226158 | 0 | -8.37343 | -5.39803 |
| 12 | Pentoxifylline | -2.214 | 3 | -7.06696 | -12.2658 | 0 | -22.7938 | -17.1916 |
| 13 | Ribavirin | 0.769 | 4 | -8.01227 | -1.14867 | 3 | -24.8383 | -11.7976 |
| 14 | sofosbuvir | -3.723 | 4 | -7.28494 | -1.07579 | 2 | -17.3142 | -23.9281 |
| 15 | tenofovir | -1.086 | 4 | -5.11416 | 3.05627 | 1 | -29.2483 | 1.95096 |

* Represents the reference standard inhibitor of FIPV-Mpro. BBB: blood brain barrier; PPB: plasma protein binding

**Figure S1:** 2D interaction pattern of the first top hit of compound Sofosbuvir complex with FIPV-Mpro


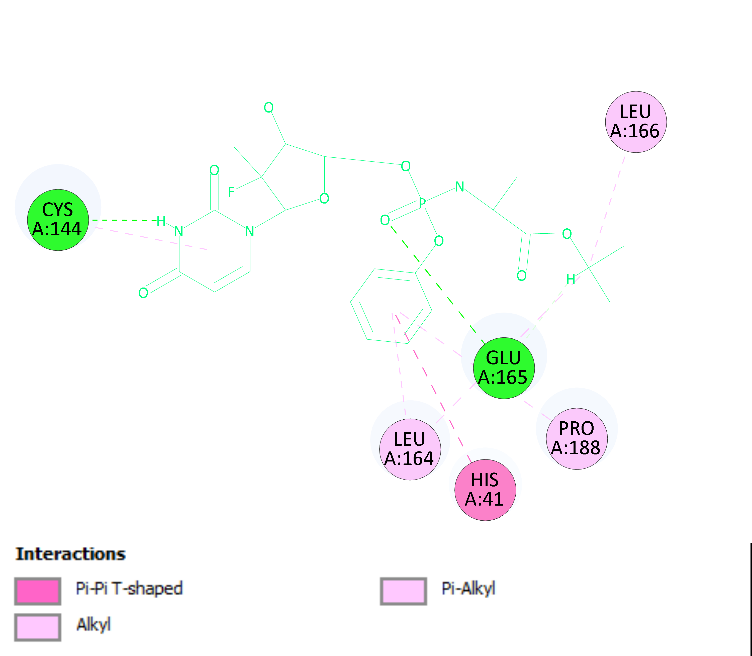


**Figure S2:** 2D interaction pattern of the first top hit of compound GS-441524 complex with FIPV-Mpro


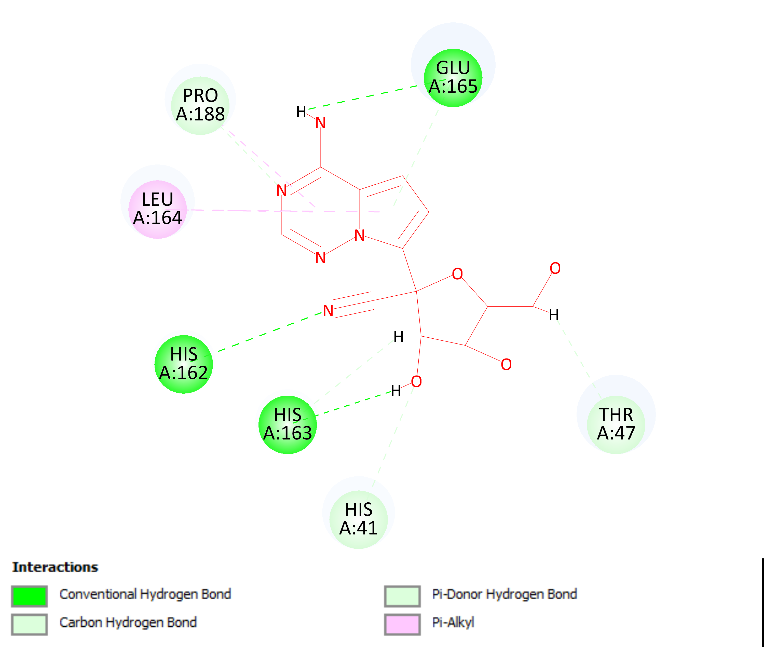


**Figure S2:** 2D interaction pattern of the top hit of compound standard N3 complex with FIPV-Mpro


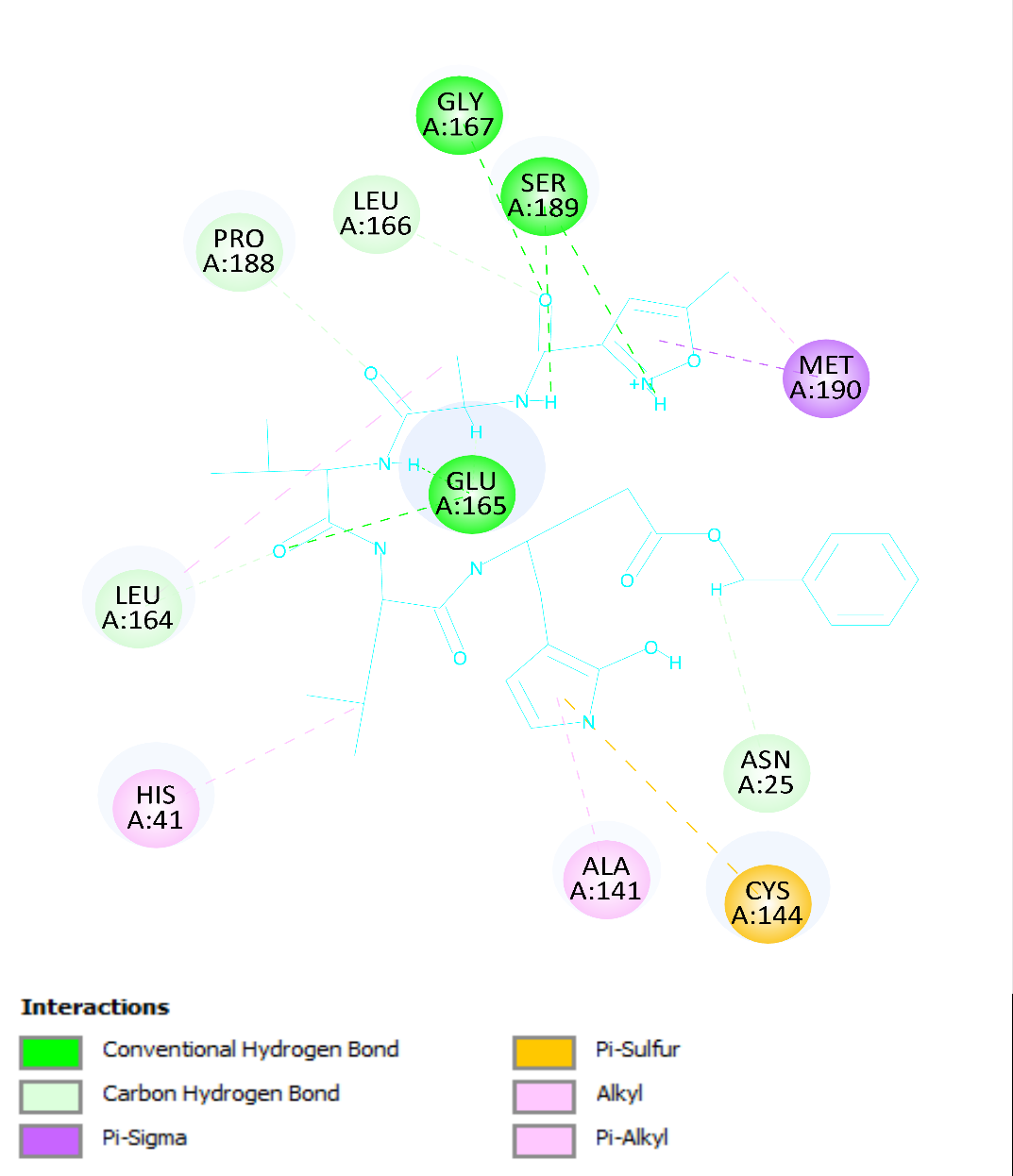


**Figure S4:** The image represents the best dock pose or top hit of compound Methotrexate for Mpro protein. (A) Mpro-Methotrexate complex after docking. (B) and (C) showing Methotrexate interaction with residues of Mpro. (D) Interaction of Methotrexate with residues including HIS41, HIS163, LEU164, GLN187, GLY167, CYS144, LEU27, VAL26 and GLU165 represented in 2-D diagram.


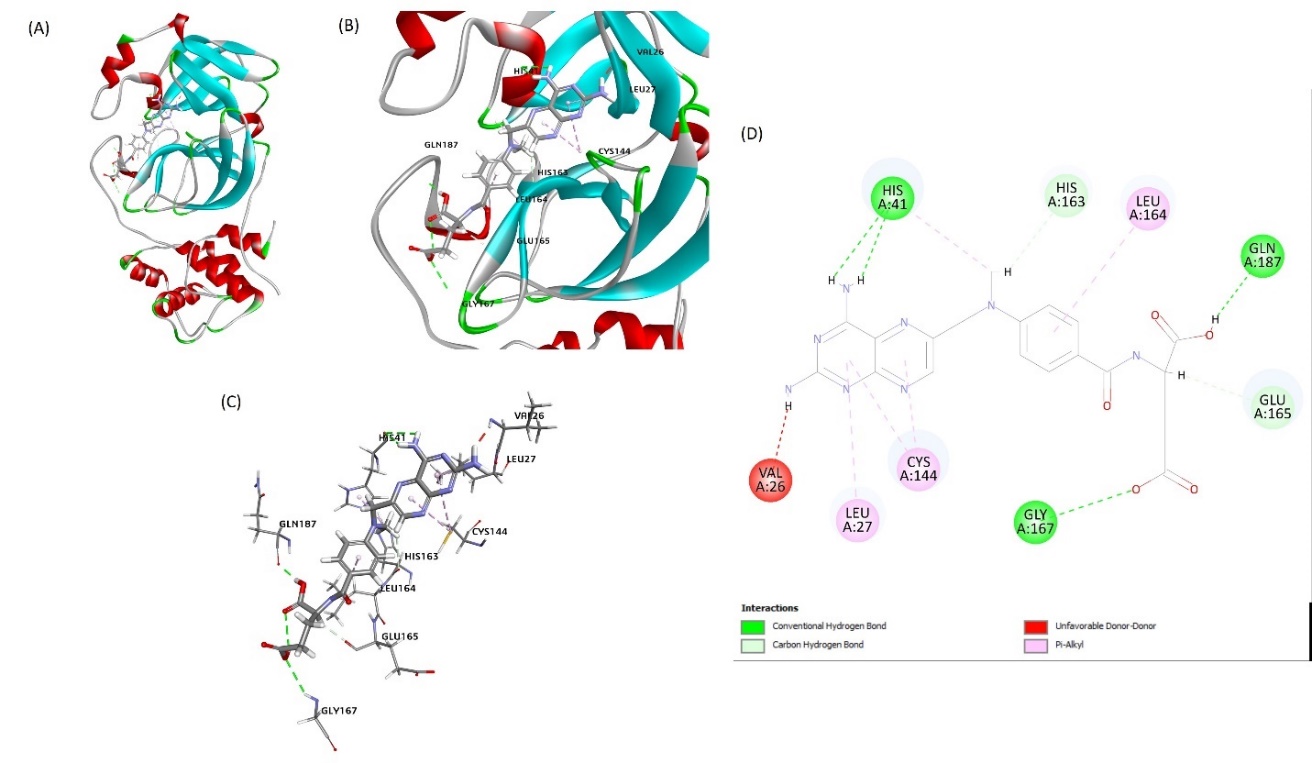


**Figure S5: The best docking pose or top hit of the interaction of the FIPV-Mpro/Pentoxifylline** interaction (A) Mpro-pentoxifylline complex after docking. (B) and (C) showing pentoxifylline interaction with residues of Mpro. (D) Interaction of pentoxifylline with FIPV-Mpro residues, including (HIS41, PRO188, LEU164, and GLU165).

**
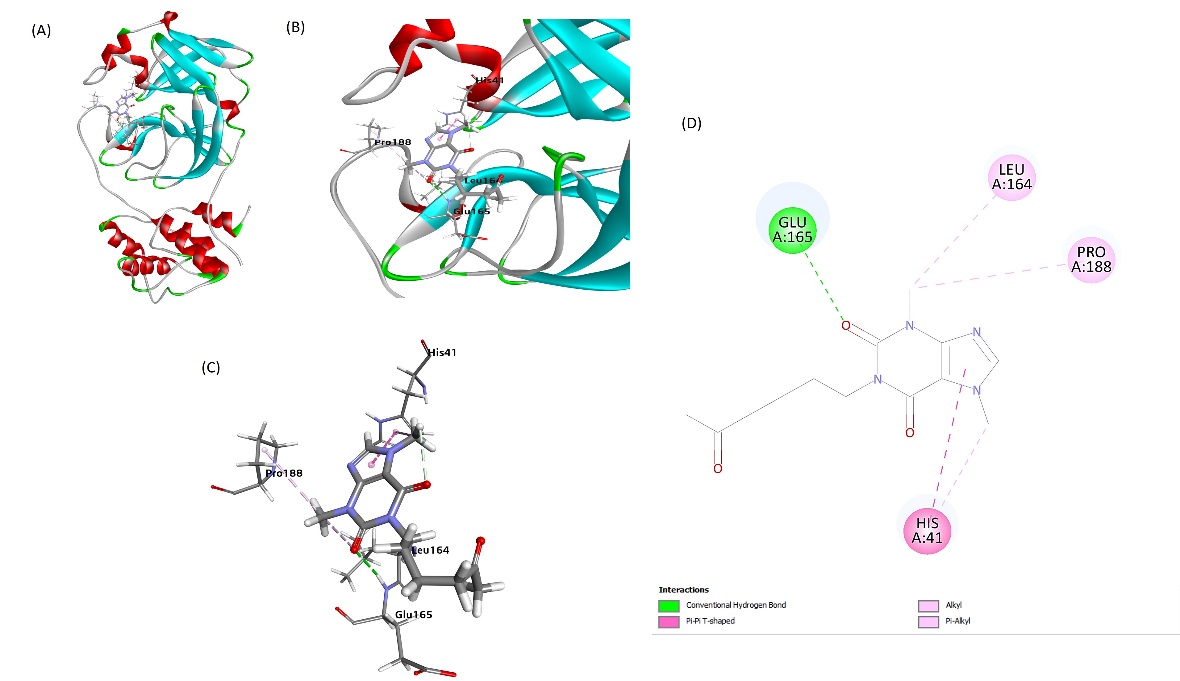
**

**Figure S6:** The image represents the best dock pose or top hit pose of compound Molnupiravir for Mpro protein. (A) Mpro-Molnupiravir complex after docking. (B) and (C) Molnupiravir interaction with residues of Mpro. (D) Interaction of Molnupiravir with residues including CYS144, LEU164, PRO188 and GLU165 represented in 2-D diagram.


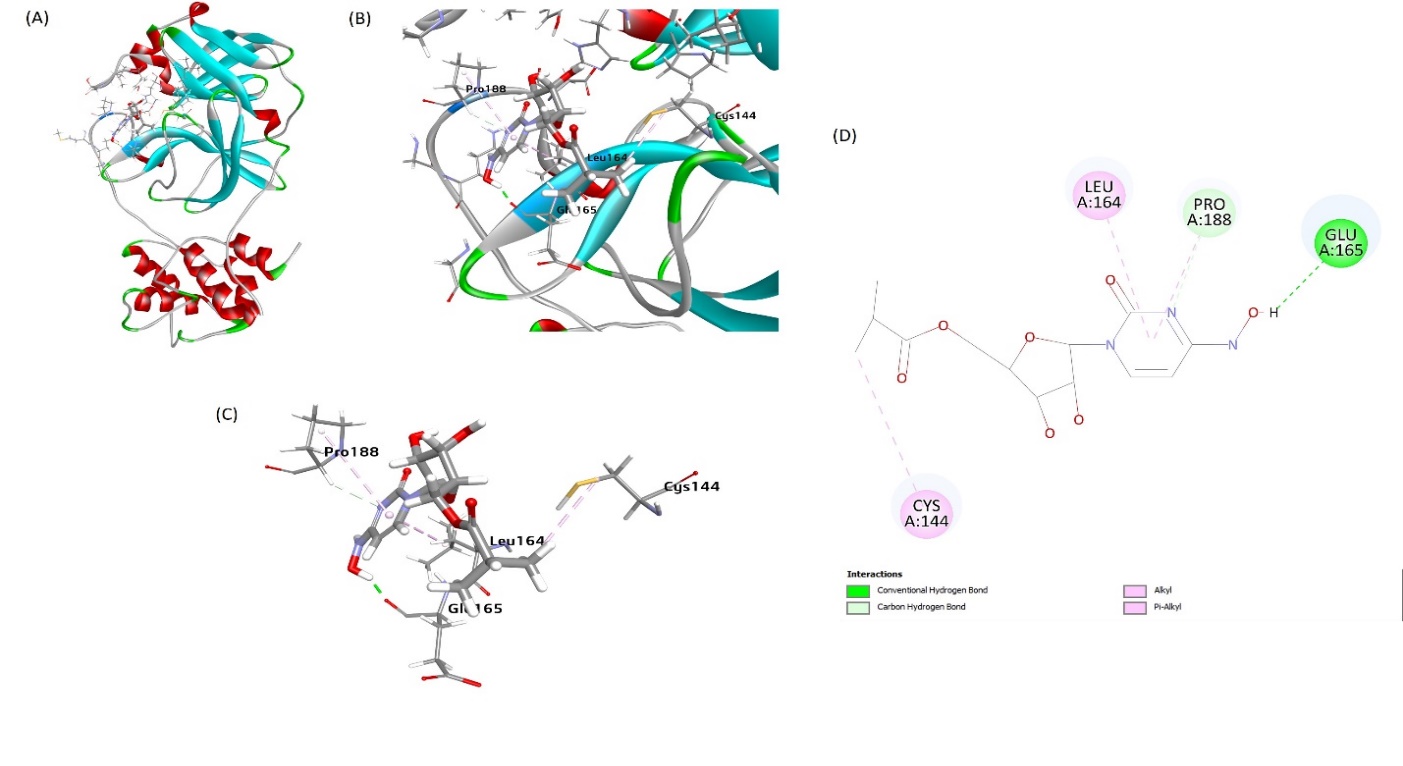

Supplement: Supplemental Information 1 [file peerj-13-19744-s001.docx]
